# Co-Occurring Alcohol Use Disorder and Anxiety: Bridging Psychiatric, Psychological, and Neurobiological Perspectives

**DOI:** 10.35946/arcr.v40.1.03

**Published:** 2019-12-30

**Authors:** Justin J. Anker, Matt G. Kushner

**Affiliations:** Justin J. Anker, Ph.D., is an assistant professor in the Department of Psychiatry, University of Minnesota, Minneapolis, Minnesota; Matt G. Kushner, Ph.D., is a professor in the Department of Psychiatry, University of Minnesota, Minneapolis, Minnesota

**Keywords:** alcohol, anxiety, comorbidity, negative affect, stress

## Abstract

A substantial number of people who have problems with alcohol also experience strong anxiety and mood problems. This article provides an overview of the evolving perspectives of this association in the context of three related disciplines—psychiatry, psychology, and neuroscience. Psychiatric and epidemiological studies show that having either an anxiety- or alcohol-related diagnosis elevates the prospective risk for developing the other disorder. From the psychological perspective, behavioral research demonstrates that drinking to cope with negative affect is a potent marker for current and future problems with alcohol. Neuroscientific research implicates overlapping neurobiological systems and psychological processes in promoting the rise of negative affect and alcohol misuse. The psychiatric perspective that alcohol misuse and co-occurring anxiety represent neurobiologically distinct diagnostic conditions has dominated the field for many decades. However, recent research provides increasing support for the neuroscientific perspective that these conditions share underlying, mutually exacerbating, neurobiological processes.

## Introduction

“Those who cannot remember the past are condemned to repeat it.”

—*George Santayana*

Few observations in psychiatry have been documented as long and as consistently as the association between anxiety (and general negative affect) and the chronic misuse of alcohol. Research has shown that up to 50% of individuals receiving treatment for problematic alcohol use also met diagnostic criteria for one or more anxiety disorders.^1^,^2^ This percentage can be compared with the prevalence of current (within the past 12 months) anxiety disorders in the U.S. community, which is estimated to be 11%.^3^,^4^

The psychiatric, psychological, and neuroscientific disciplines have developed theories to explain the association between alcohol and anxiety disorders. Each discipline has independently contributed to the understanding of how to best describe and treat alcohol use disorder (AUD) in the context of negative affectivity. However, very little cross-communication has occurred among these disciplines. This insularity and particularism continue to impose significant opportunity costs in this field.

A key challenge to applying a comparative perspective across disciplines and time is the use of unique and evolving terminology and definitions for similar phenomena. Terms such as anxiety, anxiety disorder, depression, mood disorder, tension, stress, stress disorder, and negative affect are used differently across disciplines and time. The relationships among these constructs can be conceptualized as a Venn diagram, with the shared spaces representing overlapping constructs. In these overlapping spaces, the greatest opportunities for integration across disciplines can be found. In this review, the term “negative affect” (i.e., negative hedonic tone and the biology that underpins it) describes the shared psychological and biological space for related constructs of anxiety, tension, stress-responding, and anxiety disorder.

First, historical trends and research related to the psychiatric classifications of alcohol misuse, negative affect, and their co-occurrence are reviewed, including typologies and diagnoses. Next, a history of behavioral examinations of negative affect and alcohol misuse is presented from the psychological perspective, along with a discussion of research on the use of alcohol to cope with negative affect. Finally, neurobiological research on the relationship between negative affect and alcohol use is reviewed, and the opponent process model is explained. The concluding section synthesizes the discipline-specific research to identify conclusions and unanswered questions about the connections between alcohol use and negative affect.

## Psychiatric Disorder Classifications and Diagnoses

Typologies are the oldest formal approach to categorizing alcohol misuse accompanied by strong negative affect. Summarizing dozens of such typologies from the past 200 years, Babor observed that virtually all identified an anxious-depressed subtype (Apollonian) and a revelry-oriented, rule-breaking subtype (Dionysian).^5^ The promulgation of these typologies occurred primarily in the “prescientific” era (before the 1940s), but their legacy remains evident today.

For example, Cloninger described a model in which heritable personality traits set the stage for the development of Type I or Type II “alcoholism.”^6^,^7^ Type I included people whose problems with alcohol use began later in adult life, often contemporaneous with increasing negative affect or stressful life experiences. These individuals were characterized as shy, anxious, and pessimistic (Apollonian), and their alcohol use was believed to be motivated by an effort to cope with the unpleasant subjective experiences associated with these traits. Type II included people whose problems with alcohol use began early in adult life, without reference to environmental conditions or fluctuations in internal emotional states. These individuals were characterized as having relatively less fear and guilt while engaging in relatively more rule-breaking and antisocial behavior (Dionysian), often including drinking alcohol and other drug use. Past and present typology approaches share the view that negative affect is not a separate, co-occurring condition but rather an inherent trait of a significant subtype of people who have problems with alcohol.

### Comorbidity paradigm

By the middle of the 20th century, medically oriented researchers increasingly attempted to categorize and quantify psychopathological and medical conditions observed among people being treated for the chronic misuse of alcohol.^8^ Unlike earlier typologies in which strong negative affect was considered an inherent trait of a subtype of people who had problems with alcohol, this descriptive, medical approach viewed strong anxiety and other psychiatric problems as distinct, diagnosable conditions that often co-occur with alcohol-related conditions. This conceptualization led to co-opting the medical term “comorbidity” to indicate the presence of two or more distinct psychiatric disorders.^9^ The psychiatric paradigm of comorbidity was first fully realized and codified nearly 40 years ago in the third edition of the *Diagnostic and Statistical Manual of Mental Disorders* (DSM).^10^ In the more recent DSM-5, the paradigm remains the standard psychiatric model for describing, characterizing, and treating co-occurring negative affect and AUD.^11^

### Epidemiology of co-occurring disorders

Within the co-occurring psychiatric disorder (comorbidity) paradigm, and armed with the DSM’s observable and reliable diagnostic criteria, several large, epidemiological surveys have quantified the relative risk for an alcohol-related diagnosis in the presence versus absence of a diagnosed anxiety disorder. The largest and most comprehensive community-based surveys in the United States include the Epidemiologic Catchment Area study (*N* ~ 20,000), the National Comorbidity Survey (*N* ~ 8,000), and the National Epidemiologic Survey on Alcohol and Related Conditions (NESARC, *N* ~ 43,000).

#### Alcohol-related diagnoses

An important issue in interpreting epidemiological findings is the diagnostic definition of AUD. The DSM-IV included two separate alcohol-related diagnoses: alcohol abuse and alcohol dependence.^12^ A DSM-IV diagnosis of alcohol abuse required a maladaptive pattern of ongoing drinking resulting in multiple impairments. Some impairments that met the criteria were: not fulfilling major obligations at work, school, or home; using alcohol while driving or in other physically dangerous situations; having recurrent legal problems from driving under the influence, fighting, or other actions related to alcohol use; and experiencing exacerbation of interpersonal problems because of continued alcohol use.

A DSM-IV diagnosis of alcohol dependence required meeting at least three of seven criteria.^12^ The first two criteria were physical—development of tolerance to alcohol and development of withdrawal symptoms. The remaining five criteria were behavioral signs of dependence, such as spending a great deal of time obtaining, drinking, or recovering from the effects of alcohol and drinking more alcohol, or for longer, than intended.

In the DSM-5, however, alcohol abuse and dependence have been integrated into a single diagnosis of AUD with mild, moderate, or severe subclassifications.^11^ The separate classifications of alcohol abuse and alcohol dependence were removed.

Most available epidemiological studies used diagnostic criteria from DSM-IV or earlier, and they uniformly showed a positive association between anxiety or mood disorders and alcohol dependence but not alcohol abuse. A synthesis of the major epidemiological studies showed the risk (odds) for meeting diagnostic criteria for alcohol dependence more than doubled (*OR* = 2.3) among individuals with an anxiety disorder compared to those with no anxiety disorder.^13^ However, the odds of receiving a diagnosis of alcohol abuse alone were about the same for individuals with or without an anxiety disorder (*OR* ~ 1). These results suggest that the association between anxiety disorders and AUD will diminish in forthcoming epidemiological findings (e.g., in results from the NESARC III) that use the DSM-5 diagnosis criteria.

#### Anxiety disorder diagnoses

Parallel to the question of how the definitions for alcohol-related diagnoses affect the magnitude of the association with anxiety disorders is the question of how the definitions for anxiety disorders affect that association. An early analysis^14^ of research on co-occurring disorders in the 10 years following the introduction of DSM-III criteria reached the provisional conclusion that each major subtype of anxiety disorder (i.e., social phobia disorder, panic disorder, and generalized anxiety disorder)^10^ had a unique relationship to alcohol misuse, presumably because of distinct neurobiology and symptom manifestations (e.g., discrete symptom triggers, omnipresent symptoms, or random symptom episodes). This conclusion fit neatly within the zeitgeist of that era, which presumed important clinical and biological distinctions for all psychiatric diagnoses.^10^,^13^

However, restricting attention to a single diagnosis and its relationship to alcohol misuse does not align with more recent research. For example, it is now better understood that various anxiety disorder subtypes are commonly present in the same individual.^15^,^16^ Therefore, conclusions based on epidemiological findings that focused exclusively on one anxiety disorder diagnosis without accounting for the likely presence of additional anxiety subtypes have become suspect. Also, the conclusion that each anxiety disorder subtype has a unique association with alcohol misuse is inconsistent with research showing that all the subtypes individually confer a similar increase in risk for alcohol misuse,^13^ and that the risk increases substantially for each additional anxiety disorder subtype.

Recent “big data” modeling approaches have advanced the understanding of epidemiological data related to the association between anxiety disorder subtypes and risk for alcohol misuse. Seminal work using this approach comes from Krueger, who applied structural equation modeling of latent variables related to anxiety and depression diagnoses.^17^ This research showed that a large proportion of the covariation in anxiety or mood disorder diagnoses could be characterized along a single continuum called “negative emotionality.” However, some of the variance of specific anxiety disorders was distinct from the negative emotionality continuum; that is, some variance was unique to a specific anxiety disorder subtype.

Kushner and colleagues applied this analytic approach to NESARC data to assess the relationship between risk for alcohol misuse and the shared versus unique components of several anxiety and depressive disorders.^18^ This analysis showed a strong positive relationship between risk for DSM-IV alcohol dependence and the shared components of the anxiety and depression diagnoses. However, the analysis also showed virtually no relationship between risk for alcohol dependence and the unique components of those diagnoses. These findings are inconsistent with the idea that each anxiety disorder has a unique association with the risk for alcohol misuse. Instead, the results suggest that all anxiety and mood disorders contribute to general negative emotionality, which, in turn, correlates with the risk for alcohol dependence.

### Temporal and causal priority

The elevated risk for alcohol misuse in the presence of anxiety represents a positive correlation between these conditions. One of the co-occurring conditions could be causing the other, but a third, unmeasured factor could be causing an increased risk for both conditions. When medical conditions correlate, the search for causality commonly starts by evaluating which condition preceded the other. This approach is based on the logical truism that an effect cannot precede its cause. However, preceding conditions do not necessarily cause later outcomes—the logical fallacy called “post hoc, ergo propter hoc.” Still, studies have sought to illuminate the causal associations between the co-occurring disorders by determining which began first.^19^ This research has shown that the onset of anxiety disorders preceded alcohol misuse in up to three-quarters of the people who had both conditions,^14^ especially for those who had social anxiety disorder.^20^

Failing to clearly distinguish between temporal priority and causal priority is common in interpretation of order-of-onset studies.^20^,^21^ Since its third edition, the DSM’s hierarchical diagnostic scheme designates anxiety disorders in the presence of alcohol disorders as an alcohol-induced condition unless the anxiety symptoms presented first or persisted during a period of protracted abstinence.^11^,^12^ This approach not only risks the logical error already discussed but also risks conflating initiating factors with maintaining factors. That is, this approach ignores the possibility that alcohol misuse played some role in the initiation of anxiety symptoms that over time evolved into independent anxiety disorders. However, these logical concerns may be moot empirically, because NESARC data show that the prevalence of substance-induced anxiety and mood disorders among individuals with a diagnosed alcohol disorder is vanishingly small.^4^ Unfortunately, clinical guidelines designed to avoid mistaking substance-induced anxiety or mood problems for other anxiety or depressive disorders discourage clinicians from providing effective treatments for these conditions in people who are actively drinking or recently abstinent.^22^

### Prospective relative risk

Compared to retrospective assessments of the order of onset for co-occurring disorders, assessments of prospective relative risk (i.e., the risk for developing a condition given the presence or absence of another condition) provide more information about conferred risk. For example, people typically experience onset of social anxiety disorder before they are old enough to legally purchase alcohol, so the anxiety disorder typically precedes problems with alcohol. Therefore, retrospective assessments showing that social anxiety disorder commonly precedes problems with alcohol superficially suggest that the former causes the latter. However, this type of examination provides no information about the effects of alcohol misuse on later development of social anxiety disorder.

Prospective relative risk avoids problems related to retrospectively examining the order of onset. In a study by Kushner and colleagues, the prospective relative risk of alcohol dependence and several common anxiety diagnoses was examined among approximately 500 college students during their first year, senior year, and third postgraduation year.^21^ Although anxiety disorders were more common than alcohol dependence at all assessment years, the prospective risk for new onset of either condition in a later assessment was two to five times greater if the other condition was present at an earlier assessment. Both conditions substantially increased the prospective relative risk for developing the other.

### Effects of co-occurrence on alcohol treatment outcomes

Data show that individuals who have co-occurring anxiety or depressive disorders and alcohol-related disorders have a poor response to treatment for alcohol misuse.^23^,^24^ For example, Kushner and colleagues reported that more than twice as many participants who had alcohol-related disorders and co-occurring anxiety or mood disorders, versus participants with no anxiety or mood disorder, returned to any drinking within 4 months following intensive residential treatment for alcohol misuse (52% vs. 21%).^1^

Efforts to mitigate the deleterious effects of co-occurring anxiety disorders on alcohol treatment outcomes, as well as to illuminate causal influences between these conditions, have inspired investigations into how treatment for one co-occurring condition affects symptoms of the other condition. For example, if an anxiety disorder maintains alcohol misuse, effectively treating the anxiety should reduce alcohol use and reduce the likelihood of relapse after treatment. In one study, researchers administered paroxetine or placebo in a double-blind fashion to participants who had AUD and social anxiety disorder.^25^ They found that although the medication was clinically effective in reducing social anxiety symptoms, alcohol use severity was unchanged.

Several clinical trials have examined the effect of supplementing standard AUD treatment with a validated treatment for anxiety or mood disorders among individuals with both conditions. A meta-analysis of 15 randomized controlled trials, in which medication or cognitive behavioral therapy for co-occurring anxiety or depressive disorder was added to standard treatment for AUD, showed results similar to the paroxetine study.^25^,^26^ That is, the meta-analysis showed that conventional treatments were effective at reducing co-occurring symptoms of anxiety and depression, but they did not meaningfully improve alcohol-related treatment outcomes.

## Psychological Theories

In parallel to the evolution of the descriptive psychiatric paradigm for co-occurring disorders, early psychological researchers began studying alcohol’s tension-reducing properties in laboratory (typically animal) models.^27^ It is often forgotten (or at least ignored) that this early experimental work began as a test of Freud’s theory that alcohol misuse served as an externalized ego defense mechanism. However, the research soon developed into operant-behavioral examination of what was called the “tension-reduction hypothesis.” The hypothesis maintained that alcohol’s pharmacological properties reduced tension, and this effect resulted in escalated drinking through negative reinforcement (i.e., reward generated by diminution of a noxious stimulus). In this research, the tension was any noxious state (e.g., frustration, approach-avoidance conflicts, or pain) that elicited a subjective or physiological stress response. Many dozens of laboratory studies through the latter half of the 20th century tested the tension-reduction hypothesis. Ultimately, however, the cumulative results were deemed to be “negative, equivocal, and contradictory.”^28^

In reaction to the early experimental failures and ambiguities of the operant-behavioral tension-reduction hypothesis, psychological researchers increasingly deemphasized alcohol’s putative pharmacological effects on tension. They began to emphasize the subjective expectancies, beliefs, and motivations presumed to affect a person’s decision to drink when experiencing negative affect.^29^ Drinking to cope with negative affect was viewed as a primary drinking motive.^30^ Keeping with the tension-reduction hypothesis, these researchers did not focus on formal diagnostic categories for negative affect or alcohol misuse.^31^ However, other research has linked drinking-to-cope motives with individuals who met diagnostic criteria for co-occurring AUD and anxiety disorder.^19^

An analysis of NESARC data has demonstrated that individuals who reported using alcohol to cope with the symptoms of anxiety disorder are at increased risk for persistent alcohol dependence.^19^,^32^ In addition, people with anxiety disorders who reported drinking to cope had a fivefold increased risk for developing alcohol dependence within 3 years.^32^ People with anxiety disorders who did *not* drink to cope had virtually the same prospective risk for developing alcohol dependence as people with no anxiety disorders. Further, people with anxiety disorders who did not report any drinking to cope drank less daily than people with no anxiety disorder.

## Neurobiological Theories

Starting in the 1970s, the increasing availability of biological measures offered researchers an opportunity to study the effects of alcohol on stress-responding (and vice versa) in more refined and controlled ways. This allowed for distinctions between subjective (e.g., self-reported) and objective (e.g., serum cortisol) responses to stress, as well as between immediate stress reactivity and subsequent stress regulation. Surprisingly, distinguishing subjective and objective stress-response measures revealed little connection between the two, with the former relating more directly to predictions from the tension-reduction hypothesis.^33^ Early research on stress and alcohol used these technological advancements to test the operant tension-reduction hypothesis, albeit with mixed results.^34^

### Psychophysiological and neurobiological correlates

Beginning in the 1990s, stress-related alcohol research evolved from its roots in tension-reduction research to become a multifaceted subspecialty focused primarily on the psychophysiological and neurobiological correlates of the stress response, stress regulation, and alcohol misuse. Increasingly, this research includes examination of the long-term genetic and environmental influences on stress reactivity and regulation and their connections to the development of AUD vulnerability.

For example, Brady and Back reviewed research linking early trauma and exposure to chronic stressors with permanent dysregulation in the brain systems implicated in the pathophysiology of depression, anxiety, and addiction.^35^ Other investigators reviewed research that reported associations between alcohol dependence or genetic risk for alcohol dependence and dysregulated patterns of laboratory stress-responding.^36^,^37^ Several studies have implicated chronic alcohol misuse in the dysregulation of the stress response, which contributed to further alcohol craving and increased likelihood of relapse.^38^–^40^ These and related studies demonstrate that heritable traits associated with risk for alcohol-related disorders; as well as environmental insults such as acute trauma, chronic stress, and chronic alcohol misuse; can produce durable neurobiological and subjective stress-response changes that have been associated with the development or persistence of both AUD and anxiety disorders.

### Opponent process model

**Figure 1:**
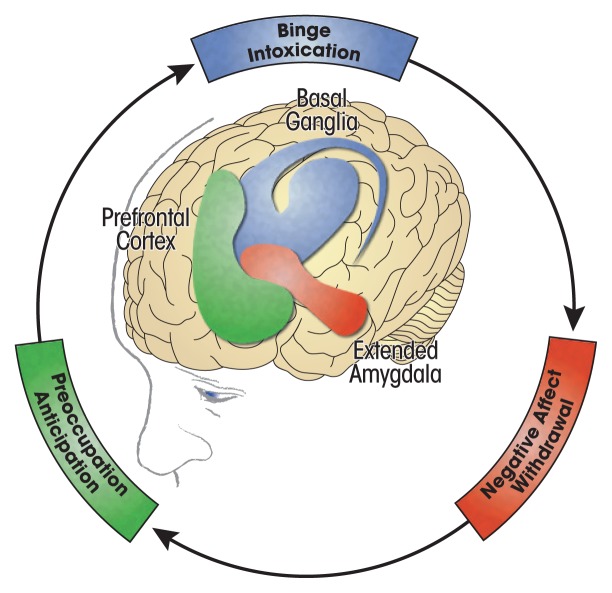
Addiction cycle stages and associated brain regions. *Source:* Adapted from U.S. Department of Health and Human Services, Office of the Surgeon General. *Facing Addiction in America: The Surgeon General’s Report on Alcohol, Drugs, and Health*. Washington, DC: U.S. Department of Health and Human Services; November 2016.

Koob and colleagues have placed both the neurobiological and subjective experiences of stress-responding and negative affect at the very center of addiction pathology (Figure 1).^41^ More specifically, they conceptualized addiction as a three-stage, pathodevelopmental cycle that engages executive function, incentive salience, and negative emotionality at different degrees during specific stages of addiction. In this opponent process model, the term “addiction” refers to the neurobiological and motivational changes that occur as a consequence of chronic substance use.

The first stage—binge/intoxication—involves activating reward circuits (e.g., the release of dopamine and opioid peptides in the ventral striatum) in response to alcohol or other drug use, which also engages incentive salience circuits.^41^ In this early stage of addiction, positive reinforcement from direct activation of the brain’s positive valence systems, as well as from formerly neutral stimuli that have become classically conditioned to evoke a pleasurable response, motivates ongoing and increased substance use. This is characterized as the impulsive stage of addiction because the goal of increasing pleasure, rather than avoiding or escaping discomfort, motivates seeking alcohol or other drugs.

In response to chronic alcohol or other drug use, both within-system and between-system brain processes seek homeostasis through dynamic, neuroregulatory, countervailing effects.^41^ However, as chronic use continues, homeostasis gives way to neuroadaptations that reset the baseline operation (allostasis) in these systems. These allostatic adaptations in the brain lead to the second stage of addiction—withdrawal/negative affect. In this stage, reward circuits become blunted because of within-system neuroadaptations. The brain’s stress systems, including corticotropin releasing factor and norepinephrine in the central amygdala and bed nucleus of the stria terminalis, become increasingly dysregulated because of between-system compensatory neuroadaptations. At this point in the addiction process, subjective negative affect predominates, especially during periods of sobriety and withdrawal. This later stage of addiction marks a shift from impulsive use driven by positive reinforcement to compulsive use driven by negative reinforcement. In this stage, compulsive substance use is aimed, in part, at decreasing the negative affect caused or aggravated by the allostatic reset in the brain’s stress and mood systems.

Finally, after these neuroadaptations have been established, the third stage of addiction—preoccupation/anticipation—undermines attempts at abstinence from drinking.^41^ At this point, chronic alcohol or other drug use becomes an integral, exogenous input for maintaining equilibrium in the brain’s mood and stress regulation systems.

Preclinical research supports the tenets of the neurobiological opponent process model.^42^ Although the model has not yet been translated to validated clinical applications, it informed the development of the Addictions Neuroclinical Assessment, a framework that uses neuropsychological data that correspond to the three stages of the neurobiological opponent process model to classify the individual differences in AUD to improve diagnosis and treatment.^43^ The model does imply specific treatment targets, such as corticotropin releasing factor^44^,^45^ and alpha_1_-noradrenergic systems.^46^ Simpson and colleagues found clinical benefit from prazosin, an alpha_1_ antagonist, in participants with an alcohol dependence diagnosis.^47^ However, the only study to examine prazosin in a sample of people with co-occurring disorders (alcohol dependence and post-traumatic stress disorder) reported that the medication had no effect on stress-responding or alcohol treatment outcomes.^48^

The opponent process model also implies that psychosocial treatments could usefully target the motive of using alcohol to cope with negative affect. Epidemiological data and the opponent process model both support the concept that this motive is a primary link between the neurobiological and subjective manifestations of negative affect and drinking behavior.^49^

## Discussion and Future Directions

The term “comorbidity” has become a fairly generic reference for co-occurring alcohol and anxiety or depressive disorders. Yet ontologically, the presence of two or more distinct, clinical diagnoses remains firmly fixed in an increasingly strained medical-diagnostic paradigm of psychopathology classification. Central to this strain is the assumption that specific diagnostic dyads are the appropriate unit of analysis for studying co-occurring negative affect and alcohol misuse. However, negative affect is common to many anxiety and depressive disorders and can increase the risk for alcohol misuse, particularly when drinking to cope with negative affect is the motive.

### Unidirectional causation theories

The notion of a simple, unidirectional, causal link between co-occurring disorders is not supported by the findings reviewed in this article. A prospective study has shown that either experiencing clinical-level anxiety or engaging in chronic alcohol misuse increases the risk of developing the other.^21^ In addition, clinical research shows that effectively treating one co-occurring condition does not substantively affect the other. Viable explanations for the relationship between co-occurring conditions include the possibility of a common cause for both conditions or bidirectional causation between the conditions. For example, dysregulated stress response or regulation may be a common risk factor for the development of both alcohol and anxiety disorders.

Also, the concept of causation among co-occurring conditions may be based on an incorrect assumption. Rather than two distinct conditions, each requiring a cause, negative affect and alcohol misuse may be parts of a single, neurobiological-behavioral syndrome. This view aligns mostly with recent neurobiological theories of addiction, but it also shares similarities with early typologies, in which negative affect was considered a fundamental trait among a large subgroup of people who had problems with alcohol.

### Shared neurobiology

The research reviewed in this article shows that trauma and chronic stress, as well as a familial risk for problems with alcohol, are associated with the dysregulated stress-response systems implicated in the development of both alcohol and anxiety disorders. In addition, chronic alcohol use is associated with dysregulated stress-responding, which, in turn, is associated with relapse following treatment for alcohol problems. Collectively, these and related findings point to overlapping neurobiological vulnerabilities.

The overlapping neurobiology of negative affect and AUD is supported by several lines of research that implicate specific brain circuits related to both conditions. The central amygdala regulates negative affect states,^45^,^50^ and research suggests the central amygdala plays a role in physiological and behavioral responses to stress, anxiety, and alcohol- or drug-related stimuli. Similarly, human imaging and animal research demonstrate abnormal central amygdala function in individuals with alcohol or anxiety disorders.^50^ A consensus is building that the central amygdala serves as a central hub for anxiety and alcohol circuits owing to its strong connection and influence on brain areas involved in executive function (medial prefrontal cortex), emotion regulation, stress responsivity (paraventricular hypothalamus and locus coeruleus), and reward processing (nucleus accumbens shell and ventral tegmental area).^45^,^50^–^53^ Crucial to the overlapping neurobiology conjecture, research shows that chronic alcohol use results in neuroadaptations to the central amygdala that are similar to the neuroadaptations that occur after chronic stress.^53^ If the neurodysregulations underlying anxiety or mood conditions and alcohol misuse overlap, it becomes reasonable to hypothesize that the common co-occurrence of these conditions may be an outgrowth of this shared neurobiology.^54^

The shared neurobiology thesis implies several unique and nonobvious hypotheses. For example, having either condition should be a risk marker for developing the other. This is consistent with prospective, observational studies showing that having either an anxiety disorder or AUD at any time increases the relative risk for future development of the other disorder. The shared neurobiology view also implies that the transition from nonproblematic alcohol use to AUD (roughly corresponding to the withdrawal/negative affect stage of addiction in the opponent process model)^41^ should require less overall alcohol exposure for people with anxiety and depressive disorders.

This hypothesis, called “telescoping,” theorizes that having either condition indicates perturbed neurobiology that is also relevant to developing the other condition. Examinations of transitions from nonproblematic or no use to problematic use of alcohol or nicotine support the telescoping hypothesis.^55^,^56^ People with anxiety disorders transitioned significantly faster than those with no anxiety disorder from initial use milestones to substance dependence. This effect was more pronounced for people who had multiple anxiety or mood disorders, even after controlling for lifetime drug exposure.^57^,^58^

### Anxiety problems in the absence of alcohol misuse

As already discussed, an analysis of epidemiological data shows that people who report drinking to cope with anxiety symptoms have increased prospective risk for developing alcohol dependence.^19^,^32^ People with anxiety disorders who do not drink to cope with their symptoms do not have an increased risk for AUD. This is good news, because most people with anxiety disorders do not report drinking to cope with their symptoms, but it also raises questions. For example, why do some people with anxiety problems drink to cope and others do not? Also, if this population has no increased risk for AUD, how is that consistent with the shared neurobiology thesis? Perhaps currently unknown factors—cultural, psychological, or biological—protect these biologically vulnerable individuals by discouraging drinking to cope.

### Alcohol misuse in the absence of anxiety

Not all people struggling with alcohol problems meet diagnostic criteria for anxiety disorders. As already discussed, an analysis of epidemiological data suggests that a DSM-IV diagnosis of alcohol abuse (i.e., negative consequences from alcohol use) without alcohol dependence does not correlate with anxiety disorder diagnoses.^13^ The opponent process model suggests that all advanced cases of substance use disorder ultimately involve negative affect (although they may not necessarily manifest as diagnosable anxiety disorders), whereas the typology and medical/diagnostic models suggest that only a particular subgroup of people who have problems with alcohol will have the key feature of negative affect.

These different models are not necessarily irreconcilable when considering the patho-developmental trajectory of addiction. During the early binge/intoxication (impulsive) stage of addiction, the opponent process model would anticipate low levels of negative affect, but during the later stage of negative affect/withdrawal, the model specifies the presence of significant negative affect and drinking to cope. Cross-sectional snapshots of people who have significant alcohol problems might reveal groups with anxiety (Apollonian) and groups without anxiety (Dionysian), but, ultimately, all may become Apollonian types as addiction advances. People who manifest anxiety problems before alcohol problems may transition very rapidly (telescope) from binge/intoxication (Dionysian) to negative affect/withdrawal (Apollonian), whereas others may make this transition more slowly or, perhaps, never.

### Stress reactivity and regulation

Stress responses in terms of both reactivity and regulation include frequently disjunctive, subjective and objective indicators. Curiously, subjective indicators of acute stress response commonly are elevated in individuals who have anxiety or alcohol problems, whereas the objective indicators tend to be acutely blunted, with diminished regulation.^58^,^59^ Also, research has well-established that perturbations in the neurobiological systems that govern biological responses to stress are associated with poorer alcohol and other substance use disorder treatment outcomes.^38^,^53^

For investigators seeking to bridge the multiple disciplines included in this review, the findings concerning stress responses pose challenges and opportunities for future research. For example, can individuals with AUD be distinguished meaningfully based on objective stress reactivity and regulation indicators, and do subjective anxiety symptoms mark or moderate this distinction? For augmenting treatment for AUD, would targeting biological stress reactivity (e.g., hypothalamic pituitary adrenal activation) be more promising than targeting anxiety disorders? Among people who have problems with alcohol, do those with versus those without co-occurring anxiety disorder react differently to protracted abstinence and withdrawal in terms of severity and persistence of dysregulation of the stress response? Prospective studies across the distinct stages of treatment and recovery for alcohol-related disorders may shed needed light on the relationships between alcohol, anxiety, and stress reactivity and regulation. Such studies have the potential to reveal the trajectory of re-regulation of the stress response during abstinence and how it relates to anxiety symptoms and relapse risk. Understanding these parameters could make a valuable contribution toward using the stress system as a recovery biomarker.

### Limitations

This review of literature from multiple disciplines required sacrificing depth for breadth. The material cited is largely limited to seminal studies and other reviews. In addition, complex research on stress and neurobiology is discussed in ways sufficient to make particular points but without providing a comprehensive or in-depth description of the underlying work. Doing so is beyond the scope of this article, but the approach presented in this article runs the risk of oversimplifying complex topics and obscuring relevant details. Also, this review does not address potentially important individual differences, such as sex.

Finally, the assumption that common areas of construct space exist across the disciplines of psychiatry, psychology, and neuroscience is open to debate. For example, medically oriented researchers might view subclinical negative affect as qualitatively rather than quantitatively distinct from diagnosed anxiety disorders. Similarly, it could be argued that dysregulated biological stress responses share little construct space with subjective negative affect and drinking to cope. However, as already noted, a dysregulated stress response is a known biological marker for the development of anxiety disorders and AUD, as well as for relapse.

## Conclusion

This review broadens the psychiatric perspective on the association between diagnosable alcohol and anxiety disorders to include the psychological/learning and neuroscientific disciplines. Cross-referencing and reconciling (if not integrating) discipline-specific approaches may reveal opportunities for synergy.

The opponent process model offers a uniquely suitable framework for transdisciplinary cross-referencing and integration. This neurobiological model aligns with the Research Domain Criteria^60^ framework’s approach to characterizing psychopathology and, thereby, avoids being trapped by the diagnostic specificity that has failed to survive empirical scrutiny. In this model, the roles of motivation and reinforcement in fundamental learning processes, which were first explored in the operant-behavioral tension-reduction hypothesis, are integrated within a pathodevelopmental framework for substance misuse. The model also accommodates individual differences in neurosusceptibility to AUD within brain systems known to be affected by stress, anxiety, and depression. To better evaluate how negative affect is associated with alcohol misuse, the opponent process model expands the scope from a narrowly defined subset of individuals with co-occurring alcohol and anxiety disorder diagnoses to include the wider range of individuals who have advanced to the negative affect/withdrawal stage of addiction. Finally, the model provides promising and specific neurobiological (e.g., corticotropin releasing factor) and psychological (e.g., drinking to cope) targets for novel interventions.
